# Supplementary material for: The effect of temperature on the boundary conditions of West Nile virus circulation in Europe
Source: PLoS Negl Trop Dis. 2024 May 6;18(5):e0012162. doi: 10.1371/journal.pntd.0012162 (PMC11098507; doi:10.1371/journal.pntd.0012162)
Supplement: S1 Text — (DOCX) [file pntd.0012162.s001.docx]

## Supporting Information S1 Text

We obtained human reported cases for the European Union from the European Surveillance System (TESSY). In the Nomenclature of Territorial Units for Statistics (NUTS) 2 regions of Utrecht (NL31), Berlin (DE30), Brandenburg (DE40), Andalucía (ES61), Veneto (ITH3), Emilia-Romagna (ITH5), Kentriki Makedonia (EL52), 1443 (58%) out of the total of 2487 human West Nile fever cases were reported to TESSY between 2010 and 2021 (Fig S2.1 and S2.2).

**
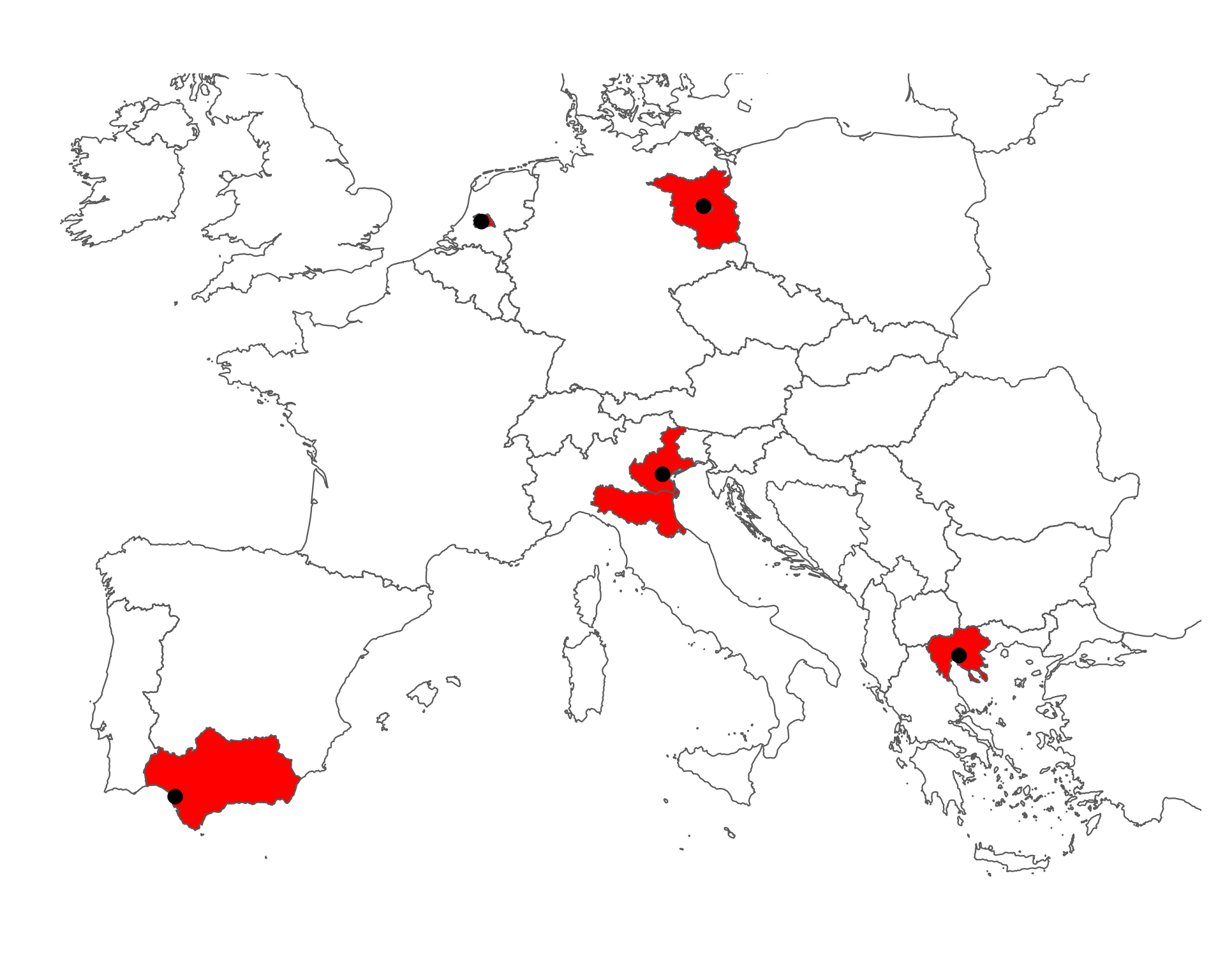
**

**Fig S1.1:** Location of the selected locations (points) and highlighted the surrounding Nomenclature of Territorial Units for Statistics (NUTS) 2 regions of Utrecht (NL31), Berlin (DE30), Brandenburg (DE40), Andalucía (ES61), Veneto (ITH3), Emilia-Romagna (ITH5), Kentriki Makedonia (EL52). Base layer maps are provided by Natural Earth under CC0 license (https://www.naturalearthdata.com/about/terms-of-use/).


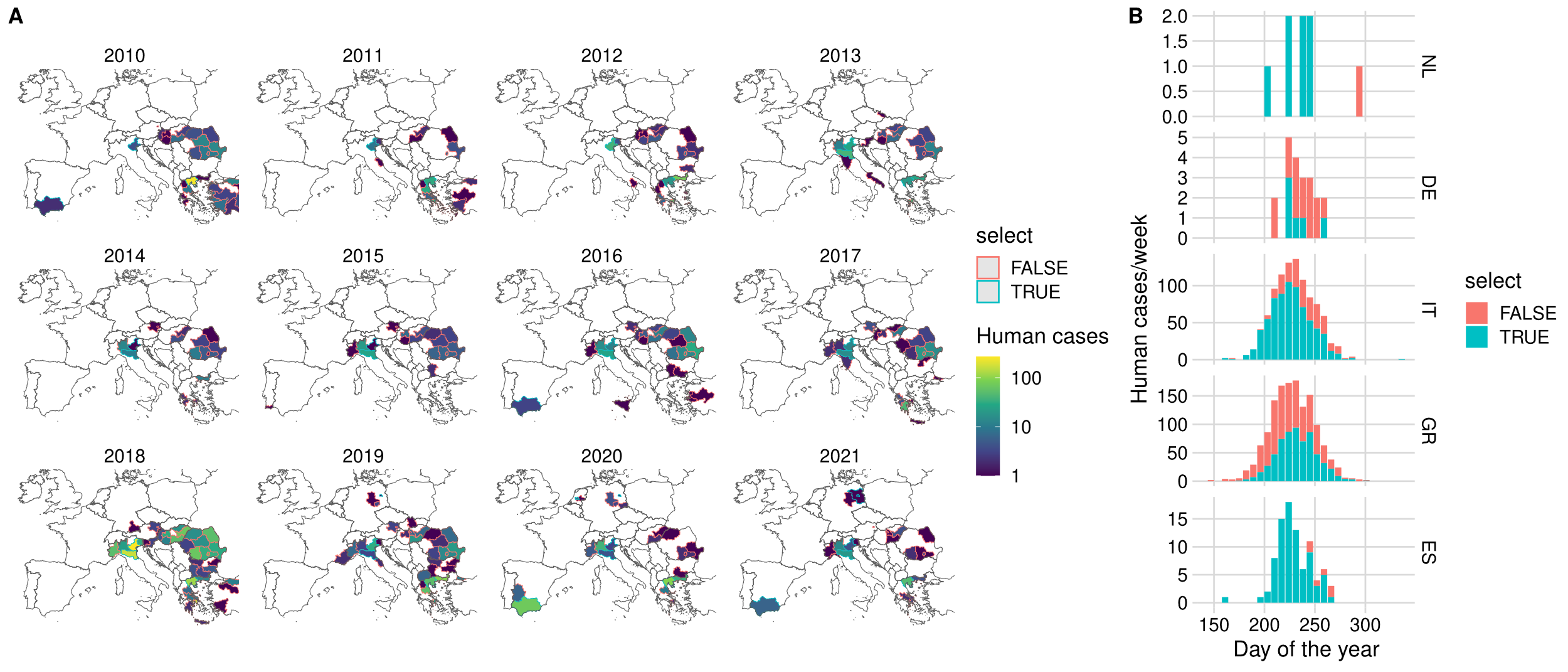


**Fig S1.2:** A. B. Cumulative number of weekly human West Nile cases for the period 2010-2021 for the five countries. Colour indicates the proportion that was in the NUTS2 regions for which we assessed the boundary conditions. The Netherlands [NL], Germany [DE], Italy [IT] and Greece [GR], Spain [ES]. Base layer maps are provided by Natural Earth under CC0 license (https://www.naturalearthdata.com/about/terms-of-use/).
