# Supplementary material for: The effect of temperature on the boundary conditions of West Nile virus circulation in Europe
Source: PLoS Negl Trop Dis. 2024 May 6;18(5):e0012162. doi: 10.1371/journal.pntd.0012162 (PMC11098507; doi:10.1371/journal.pntd.0012162)
Supplement: S2 Text — (DOCX) [file pntd.0012162.s002.docx]

## Supporting Information S2 Text

Equation S1 (quadratic) and S2 (Briére) are functions of temperature (*T*). These depend on a lower and upper thermal limit (*T_min,_,T_max_*) and a coefficient (*q*).

Quadratic: $f(T)=-q(T-T_{min})(T-T_{max})$ (S1)

Briére: $f(T)=q\cdot T(T-T_{min})\sqrt{(T_{max}-T)}$ (S2)

These functions were fit to laboratory observations where under controlled temperature regimes disease and lifecycle parameters were estimated (see Fig 2).


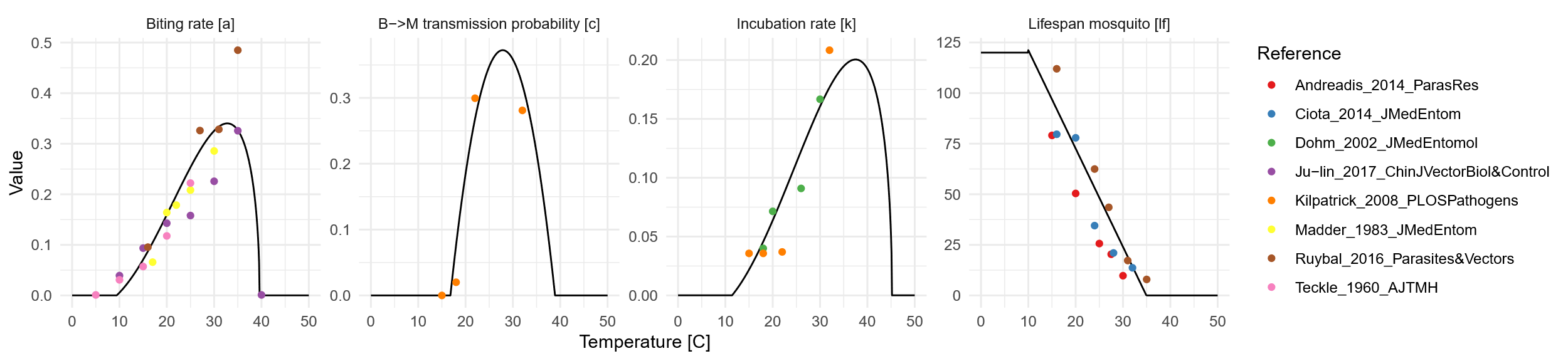


**Fig S2.1:** Temperature-response curves on mosquito biting rate (*a*, days^-1^), proportion of mosquitoes that become infectious after taking a blood meal on an infected bird (c), rate at which mosquitoes become infectious (*k* days^-1^, 1*/k* = incubation time in days), and mosquito lifespan in days (*lf*, *µ_A_* = 1/*lf*).
