# Supplementary material for: The effect of temperature on the boundary conditions of West Nile virus circulation in Europe
Source: PLoS Negl Trop Dis. 2024 May 6;18(5):e0012162. doi: 10.1371/journal.pntd.0012162 (PMC11098507; doi:10.1371/journal.pntd.0012162)
Supplement: S3 Text — (DOCX) [file pntd.0012162.s003.docx]

## Supporting Information S3 Text

To assess the West Nile virus (WNV) prevalence in mosquitoes in endemic regions in Spain, Italy and Greece, we collected data from literature. We included studies where the presence of WNV was assessed in *Cx. Pipiens* mosquitoes, and that were conducted from 2010 until 2022, the year of the literature search. We identified 16 studies that qualified these inclusion criteria [1-16]. In these studies mosquitoes were trapped and pooled in groups of 50-200 mosquitoes, and occasionally in smaller pools. We extracted the number of positive pools, the number of pools tested, the pool size and the total number of mosquitoes tested. We calculate both the minimum infection rate (MIR), as the ratio of the number of positive pools (Y) to the total number of mosquitoes tested (t), and the MLE (Equation S1), with the number of positive pools (Y), the number of pools (X), and the pool size (m) assuming a constant pool size [17]. Since often the exact pool sizes were not reported, we assumed a constant pool size calculated by the reported number of tested mosquitoes divided by the reported number of pools tested. When infection rates are higher, these methods are known to underestimate the infection rates. Furthermore, we calculated the average weekly trapping rate by dividing the total number of trapped mosquitoes by the number of trapping weeks.

$MLE=1-{(1-\frac{Y}{X})}^{1/m}$ Eq. S1

We found in these areas that on average 1.0/1000 (MIR) or 1.2/1000 (MLE) of the trapped *Cx Pipiens* mosquitoes were positive. The median weekly trapping rate was 955 mosquitoes, but varied greatly between studies since the number of traps used varied similarly.

**Table. S1. Summary of mosquitoes studies conducted in Italy, Greece and Spain between 2010-2022.**

| **Country** | **Year(s)** | **Trapping months** | **Mosquitoes tested** | **Pools test (positive)** | **MIR^a^** | **MLE^a^** | **Trapping**  **Rate (week^-1^)** | **Reference** |
| --- | --- | --- | --- | --- | --- | --- | --- | --- |
| Italy | 2018 | 5 | 269112 | 2331 (232) | 0.86 | 0.91 | 11961 | [52] |
| Italy | 2008-2014 | 8 | 2346224 | 106 (5) | 0 | 0 | 65173 | [53] |
| Italy | 2010-2011 | 7.5 | 1156 | 131 (0) | 0 | 0 | 34 | [53] |
| Italy | 2014 | 5 | 10757 | 914 (3) | 0.28 | 0.27 | 478 | [54] |
| Italy | 2014 | 5 | 78131 | 1514 (9) | 0.12 | 0.11 | 3472 | [55] |
| Italy | 2013 | 5 | 510773 | 7256 (178) | 0.35 | 0.35 | 22701 | [56] |
| Italy | 2010-2012 | 18 | 262905 | NR (23) | 0.09 | NR | 3246 | [57] |
| Italy | 2011 | 5 | 69025 | 2732 (5) | 0.07 | 0.07 | 3068 | [58] |
| Italy | 2009-2011 | 2 | 4799 | 172 (0) | 0 | 0 | 533 | [59] |
| Italy | 2008-2011 | 5 | 818474 | 5645 (32) | 0.04 | 0.04 | 36377 | [59] |
| Italy | 2011 | 5 | 69172 | 2732 (5) | 0.07 | 0.07 | 3074 | [60] |
| Greece | 2020 | 4 | 2809 | 70 (10) | 3.56 | 3.85 | 156 | [61] |
| Greece | 2018 | 5 | 17470 | 229 (10) | 0.57 | 0.59 | 776 | [62] |
| Greece | 2010 | 2 | 6597 | 110 (2) | 0.3 | 0.31 | 733 | [63] |
| Greece | 2011 | 6 | 61812 | 824 (60) | 0.97 | 1.01 | 2289 | [63] |
| Greece | 2012 | 5 | 87643 | 663 (98) | 1.12 | 1.21 | 3895 | [63] |
| Greece | 2013 | 5 | 3906 | 130 (44) | 11.26 | 13.68 | 174 | [63] |
| Greece | 2013-2013 | 6 | 25780 | 295 (9) | 0.35 | 0.36 | 955 | [64] |
| Greece | 2010-2011 | 8 | 16116 | 296 (6) | 0.37 | 0.38 | 448 | [65] |
| Greece | 2010 | 1 | 3232 | 65 (2) | 0.62 | 0.62 | 718 | [66] |
| Spain | 2020 | 6 | 1563 | 152 (1) | 0.64 | 0.66 | 58 | [67] |

^a^ minimum infection rate (MIR) and maximum likelihood estimation (MLE) of the infection rate per 1000 mosquitoes. NR = not reported/unable to calculate due to missing data.

### References S3 Text

1. Calzolari M, Angelini P, Bolzoni L, Bonilauri P, Cagarelli R, Canziani S, et al. Enhanced West Nile Virus Circulation in the Emilia-Romagna and Lombardy Regions (Northern Italy) in 2018 Detected by Entomological Surveillance. Front Vet Sci. 2020;7:243. Epub 20200505. doi: 10.3389/fvets.2020.00243. PubMed PMID: 32432132; PubMed Central PMCID: PMCPMC7214930.

2. Mancini G, Montarsi F, Calzolari M, Capelli G, Dottori M, Ravagnan S, et al. Mosquito species involved in the circulation of West Nile and Usutu viruses in Italy. Vet Ital. 2017;53(2):97-110. doi: 10.12834/VetIt.114.933.4764.2. PubMed PMID: 28675249.

3. Pautasso A, Radaelli MC, Ballardini M, Francese DR, Verna F, Modesto P, et al. Detection of West Nile and Usutu Viruses in Italian Free Areas: Entomological Surveillance in Piemonte and Liguria Regions, 2014. Vector Borne Zoonotic Dis. 2016;16(4):292-4. Epub 20160210. doi: 10.1089/vbz.2015.1851. PubMed PMID: 26862776.

4. Ravagnan S, Montarsi F, Cazzin S, Porcellato E, Russo F, Palei M, et al. First report outside Eastern Europe of West Nile virus lineage 2 related to the Volgograd 2007 strain, northeastern Italy, 2014. Parasit Vectors. 2015;8:418. Epub 20150813. doi: 10.1186/s13071-015-1031-y. PubMed PMID: 26265490; PubMed Central PMCID: PMCPMC4534017.

5. Calzolari M, Pautasso A, Montarsi F, Albieri A, Bellini R, Bonilauri P, et al. West Nile Virus Surveillance in 2013 via Mosquito Screening in Northern Italy and the Influence of Weather on Virus Circulation. PLoS One. 2015;10(10):e0140915. Epub 20151021. doi: 10.1371/journal.pone.0140915. PubMed PMID: 26488475; PubMed Central PMCID: PMCPMC4619062.

6. Gobbi F, Capelli G, Angheben A, Giobbia M, Conforto M, Franzetti M, et al. Human and entomological surveillance of West Nile fever, dengue and chikungunya in Veneto Region, Italy, 2010-2012. BMC Infect Dis. 2014;14:60. Epub 20140205. doi: 10.1186/1471-2334-14-60. PubMed PMID: 24499011; PubMed Central PMCID: PMCPMC3922982.

7. Mulatti P, Bonfanti L, Capelli G, Capello K, Lorenzetto M, Terregino C, et al. West Nile virus in north-eastern Italy, 2011: entomological and equine IgM-based surveillance to detect active virus circulation. Zoonoses Public Health. 2013;60(5):375-82. Epub 20120913. doi: 10.1111/zph.12013. PubMed PMID: 22971022.

8. Calzolari M, Bonilauri P, Bellini R, Albieri A, Defilippo F, Tamba M, et al. Usutu virus persistence and West Nile virus inactivity in the Emilia-Romagna region (Italy) in 2011. PLoS One. 2013;8(5):e63978. Epub 20130507. doi: 10.1371/journal.pone.0063978. PubMed PMID: 23667694; PubMed Central PMCID: PMCPMC3646878.

9. Savini G, Capelli G, Monaco F, Polci A, Russo F, Di Gennaro A, et al. Evidence of West Nile virus lineage 2 circulation in Northern Italy. Vet Microbiol. 2012;158(3-4):267-73. Epub 20120217. doi: 10.1016/j.vetmic.2012.02.018. PubMed PMID: 22406344.

10. Papa A, Tsioka K, Gewehr S, Kalaitzopouou S, Pervanidou D, Vakali A, et al. West Nile fever upsurge in a Greek regional unit, 2020. Acta Trop. 2021;221:106010. Epub 20210612. doi: 10.1016/j.actatropica.2021.106010. PubMed PMID: 34129841.

11. Papa A, Gewehr S, Tsioka K, Kalaitzopoulou S, Pappa S, Mourelatos S. Detection of flaviviruses and alphaviruses in mosquitoes in Central Macedonia, Greece, 2018. Acta Trop. 2020;202:105278. Epub 20191120. doi: 10.1016/j.actatropica.2019.105278. PubMed PMID: 31756306.

12. Patsoula E, Vakali A, Balatsos G, Pervanidou D, Beleri S, Tegos N, et al. West Nile Virus Circulation in Mosquitoes in Greece (2010-2013). Biomed Res Int. 2016;2016:2450682. Epub 20160512. doi: 10.1155/2016/2450682. PubMed PMID: 27294111; PubMed Central PMCID: PMCPMC4880692.

13. Papa A, Papadopoulou E, Kalaitzopoulou S, Tsioka K, Mourelatos S. Detection of West Nile virus and insect-specific flavivirus RNA in Culex mosquitoes, central Macedonia, Greece. Trans R Soc Trop Med Hyg. 2014;108(9):555-9. Epub 20140717. doi: 10.1093/trstmh/tru100. PubMed PMID: 25033823.

14. Papa A, Xanthopoulou K, Tsioka A, Kalaitzopoulou S, Mourelatos S. West Nile virus in mosquitoes in Greece. Parasitol Res. 2013;112(4):1551-5. Epub 20130131. doi: 10.1007/s00436-013-3302-x. PubMed PMID: 23371497.

15. Papa A, Xanthopoulou K, Gewehr S, Mourelatos S. Detection of West Nile virus lineage 2 in mosquitoes during a human outbreak in Greece. Clin Microbiol Infect. 2011;17(8):1176-80. Epub 20110114. doi: 10.1111/j.1469-0691.2010.03438.x. PubMed PMID: 21781205.

16. Figuerola J, Jimenez-Clavero MA, Ruiz-Lopez MJ, Llorente F, Ruiz S, Hoefer A, et al. A One Health view of the West Nile virus outbreak in Andalusia (Spain) in 2020. Emerg Microbes Infect. 2022;11(1):2570-8. doi: 10.1080/22221751.2022.2134055. PubMed PMID: 36214518; PubMed Central PMCID: PMCPMC9621199.

17. Gu W, Lampman R, Novak RJ. Problems in Estimating Mosquito Infection Rates Using Minimum Infection Rate. Journal of Medical Entomology. 2003;40(5):595-6. doi: 10.1603/0022-2585-40.5.595.
