# Supplementary figures and images for: The effect of temperature on the boundary conditions of West Nile virus circulation in Europe
Source: PLoS Negl Trop Dis. 2024 May 6;18(5):e0012162. doi: 10.1371/journal.pntd.0012162 (PMC11098507; doi:10.1371/journal.pntd.0012162)

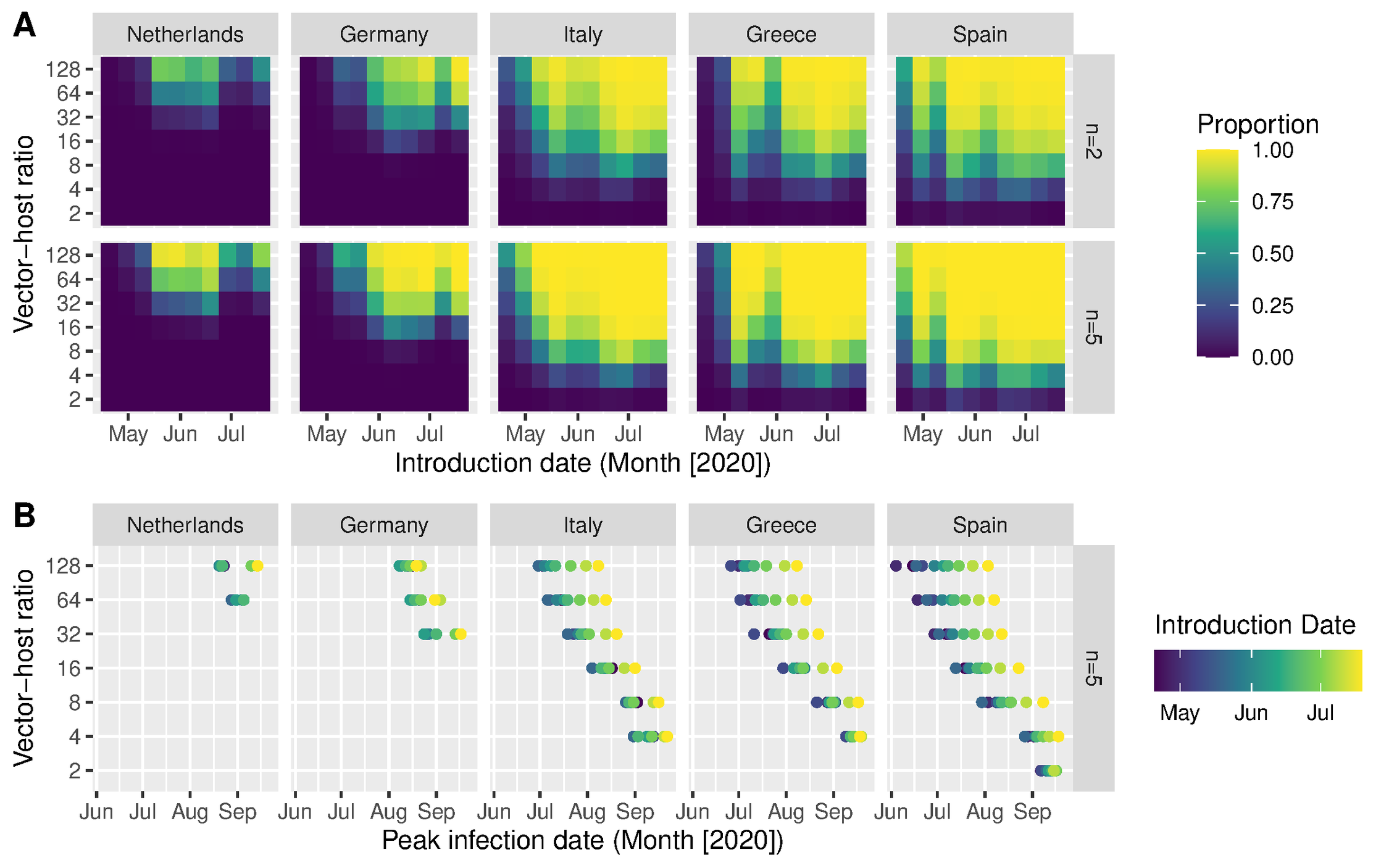

Supplement: S1 Fig — (TIF) [file pntd.0012162.s004.tif]

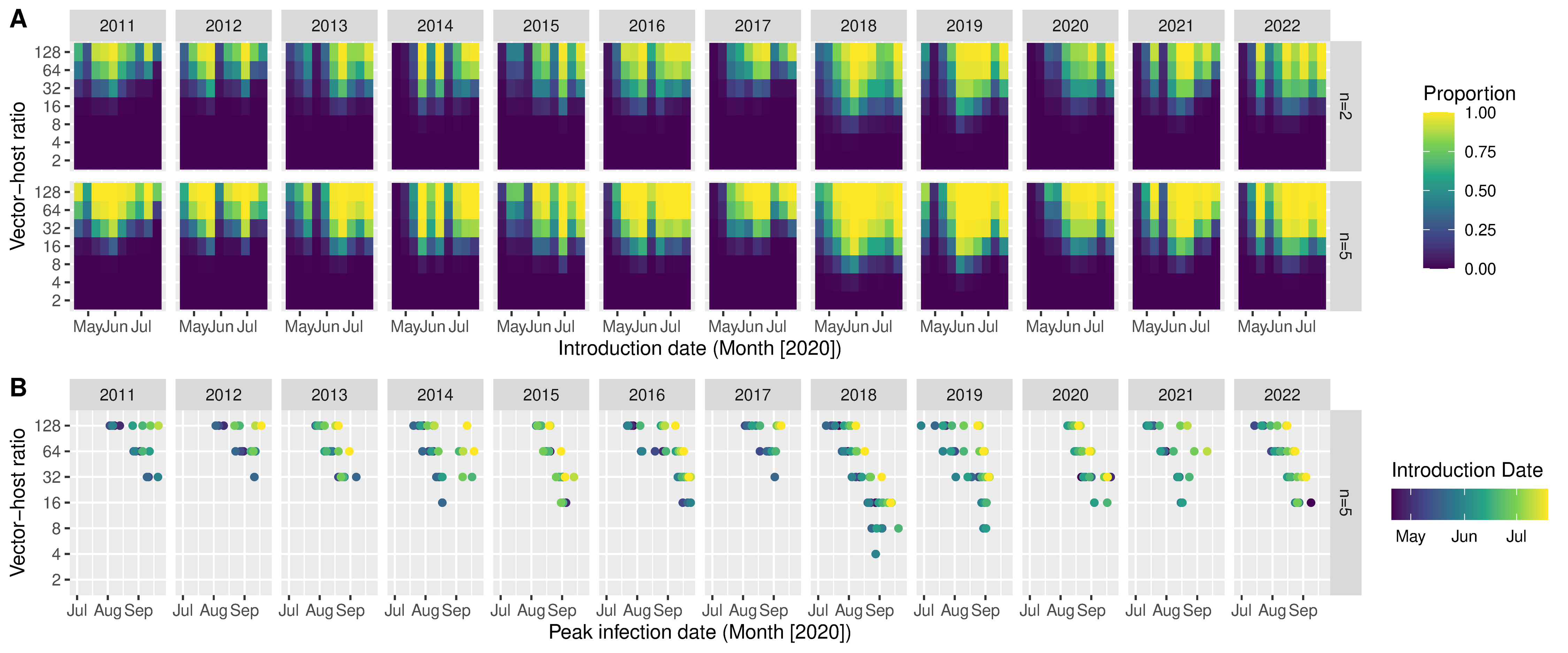

Supplement: S2 Fig — (TIF) [file pntd.0012162.s005.tif]
