# Supplementary material for: The effect of temperature on the boundary conditions of West Nile virus circulation in Europe
Source: PLoS Negl Trop Dis. 2024 May 6;18(5):e0012162. doi: 10.1371/journal.pntd.0012162 (PMC11098507; doi:10.1371/journal.pntd.0012162)
Supplement: Attachment — Submitted filename: LetterPNTD-D-23-01408.docx [file pntd.0012162.s006.docx]

Response to decision on “PLOS Neglected Tropical Diseases submission (PNTD-D-23-01408)”

Manuscript: “The effect of temperature on the boundary conditions of West Nile virus circulation in Europe”

Dear editor and reviewers,

We appreciate all comments and suggestions for our manuscript. We received it very positively, and we agree that it will improve the quality of the publication. We approached the referees’ comments separately in this letter to guide the reading of corrections and indication of the corrections in the text. There is a section for reactions to the minor comments (for both reviewers). Below we provide the answers in red [**Answer**]. Line numbers below refer to the document with tracked changes.

Documents:

- Revision_WNV_EURtempModel_cleanManuscriptv1_nosupplement_Clean.docx [version without tracked changes]
- Revision_WNV_EURtempModel_cleanManuscriptv1_nosupplement_TrackedChanges.docx [version with tracked changes]

**Reviewer #1:**

Costa de Freitas et al. modeled the effect of temperature on the boundary conditions of West Nile virus circulation in Europe. The authors used a compartmental model to explore under which conditions West Nile virus transmission can take place in five locations in Europe. These locations varied in their history of West Nile virus transmission, ranging from endemic regions with decades of reported transmission and newly emerging regions. The main findings are that temperature is an important limiting factor to West Nile virus transmission in the newly emerging regions, whereas it is hardly a limiting factor in endemic regions. This work provides important insights to better inform risk assessments and surveillance strategies.

The two main limitations of the model are the assumptions that (1) mosquito and bird populations are constant over time, and (2) vector-host ratio is constant over the season. The authors did an exceptional good job of acknowledging these limitations, which stem from lack of mosquito and bird abundance data availability, and they provide specific recommendations for how these current data gaps can be addressed in future work. Despite these limitations, the model provides important insights into retrospective transmission patterns in previous years, and the role of temperature. The only thing that in my opinion is still missing is a section in the discussion on how this model can be used/adapted to predict future transmission (i.e., can this approach be used to predict what level of transmission is expected in the following months, which can then be used to inform control strategies to prevent transmission? Or can this only be used for retrospective data?).
[**Answer**] This is a very relevant comment from a risk management perspective. We addressed this comment at the end of the fourth paragraph of the discussion section. We now offer the perspective that temperature predictions (in the near future, or with inputs from climate modelling in the further future) could provide insight in the risk of transmission. However, we also emphasize that temperature is just a part of the complex relationship between mosquito, virus and environment.

“Mosquito abundance is driven by different ecological drivers namely light intensity, CO2, temperature, humidity, housing condition, drainage, and vegetation, depending on the location and local composition of habitats, their carrying capacity and the mosquito population [18, 33, 49].”

**Reviewer #2:**

How did the authors arrive at the total number of mosquitos populating the SEI model? Other ecological factors besides temperature, e.g., precipitation, are drivers of the total number of mosquitoes that begin the transmission cycle and factor into the vector-host ratio.

[**Answer**] We have clarified that the vector-host ratios are assumed constant over time, and the exploration of the different vector-host ratios are part of the research question. Thus, there was no specific total number of mosquitoes to be identified.

**Line 221-222:** “In all implementations of the model, we assume that the vector-host ratio remains constant throughout the season.”

These are thus not influenced by ecological factors, despite the fact that it is the case in reality. We did already raise this point in the discussion:

“First, we assumed that the vector-host ratios were constant per season. In reality this is not the case, and overestimating the vector-host ratio early in the season will inflate the probability of transmission. Thus, the results presented here can be considered as ’worst-case’ scenarios, since reality will be a composite of different vector-host ratios over time. Understanding what drives mosquito and bird abundance helps to further inform vector-host ratios. However, at a European scale, data is lacking and mosquito abundance models [48] often only approach abundance as peak-abundance and fail to provide temporal changes.”

Although the model includes only temperature as a driving force for WNV transmission, it may be important to mention in the conclusion that other ecological drivers should be taken into consideration to model the total number of mosquitoes entering the model.

[**Answer**] We appreciate this comment. This is a relevant point that should be raised in the conclusion. We now mention that more factors are possible drivers for mosquito abundance, which should be explored in further model implementation.

**Line 343-345:** “Mosquito abundance is driven by different ecological drivers namely light intensity, CO2, temperature, humidity, drainage, and vegetation, depending on the location and local composition of habitats, their carrying capacity and the mosquito population [18, 33, 49].”

This also links to the previous comment, if better data becomes available to estimate the relationship at a European scale between ecological drivers and WNV transmission risk, beyond temperature, the model could be updated to reflect this knowledge.

**Minor comments**

1.	The species name should not be capitalized: e.g. Culex pipiens instead of Culex Pipiens.

[**Answer**] We appreciate this observation. The reviewer can now see that we corrected it in the whole document.

2.	Line 357: for consistency, I suggest changing “subtypes” to “biotypes”.

[A**nswer**] We replaced the word “subtypes” with “biotypes”. We further clarified that it is Culex pipiens sensu stricto (s.s.).
